# Relationship between feedback frequency and task performance: evidence on the mediating role of heart rate

**DOI:** 10.3389/fpsyg.2025.1438865

**Published:** 2025-03-26

**Authors:** Mitali Praveen Kumar Saxena, Venkat Ram Reddy Ganuthula

**Affiliations:** School of Management and Entrepreneurship, Indian Institute of Technology, Jodhpur, India

**Keywords:** HR, HRV, cognitive load, feedback frequency, task performance

## Abstract

This study looks into the impact of feedback frequency, a source of cognitive load, on heart rate (HR) and heart rate variability (HRV). The experiment was undertaken using a sample of 96 university students, wherein the feedback frequency was manipulated during an arithmetic task performance. During the task, participants were instructed to wear the E4 Empatica device, which is used to assess HR and HRV. The metrics used to measure HRV were the Standard Deviation of NN Intervals (SDNN) and Root Mean Square of Successive Differences (RMSSD). The study revealed notable disparities in the average HR values across the three feedback conditions. Optimal performance was achieved when HR was elevated (indicating the highest level of cognitive load) and feedback frequency was moderate. Further, HR mediated the association between feedback frequency and task performance. However, no significant impact of feedback frequency on HRV was found.

## 1 Introduction

Feedback is crucial in personal, professional, educational, and organizational development. Bakker (2015) suggested performance feedback as a job resource that assists individuals in maximizing the alignment between their needs, abilities, and job requirements. Feedback enhances the learning process and improves individual performance. This has been echoed by Park et al. (2019), Lam et al. (2011), and Saenz et al. (2019). Sparr and Sonnentag (2008) emphasized the importance of the feedback environment in promoting well-being in the workplace. For a review of the significance of supervisory feedback and its impact on the workplace, we refer to Dello Russo et al. (2022).

Feedback is commonly obtained from external sources but can also be obtained through self-evaluation, a less commonly studied approach (Mabe and West, 1982). To experience a sense of competence, an individual needs to possess the ability to assess their performance (Ilgen et al., 1979). However, placing exclusive reliance on self-evaluation may not consistently yield accurate information. Therefore, one must possess external manifestations to assess one’s ability (Ilgen et al., 1979). Feedback can be analyzed from different dimensions, including valence (Zhou, 1998), source (Brett and Atwater, 2001; Vancouver and Morrison, 1995), type (Sheppard, 1992), specificity (Earley et al., 1990; Goodman et al., 2004), and frequency (Chhokar and Wallin, 1984; Lam et al., 2011), the latter of which remains understudied.

One of the critical aspects of feedback is frequency, which plays a vital role in shaping performance outcomes (Lam et al., 2011). According to Ilgen et al. (1979), maintaining a balanced feedback frequency is crucial for enhancing the recipient’s sense of competence. Lam et al. (2011) recommend that a moderate frequency is preferable to optimize performance. However, some studies propose that enhancing feedback can enhance performance outcomes. For instance, the studies conducted by Bilodeau (1966) and Cook (1968) demonstrated that increased feedback on simulated tasks facilitated individuals to enhance their performance progressively. According to Anderson et al. (1971), people with the correct response knowledge demonstrated much more learning than those not provided with the same information. This highlights the need to identify the optimal feedback frequency for optimal performance outcomes.

Though prior literature has explored the relationship between feedback frequency and task performance, the underlying mechanisms driving this relationship remain unclear. To address this gap, we draw on cognitive load theory (CLT, Sweller, 2011), which suggests that feedback serves as a source of information that causes intrinsic and extraneous cognitive load. In support of this, Wang et al. (2019) found that detailed feedback resulted in lower extraneous cognitive load. Since the learners’ cognitive resources are limited, including feedback as a stimulus throughout the performance stage may significantly impact the individual’s cognitive load (Lam et al., 2011). According to Higgins et al. (1986) and Kaegi et al. (1999), self-evaluation can place a more significant burden on working memory due to the lack of feedback, leading to mental stress and a range of emotional responses. Thus, identifying the optimal feedback frequency is essential for managing cognitive load effectively and enhancing performance outcomes.

This study aims to answer the following research questions:

**RQ1:** What is the relationship between feedback frequency and cognitive load?
**RQ2:** What is the optimal feedback frequency to achieve optimal performance outcomes?
**RQ3:** How does cognitive load shape the relationship between feedback frequency and task performance?

The cognitive load causes changes in physiological factors such as heart rate (HR, Cranford et al., 2014; Mulder, 1992; Vanneste et al., 2021) and heart rate variability (HRV, Ayres et al., 2021; Solhjoo et al., 2019; Vanneste et al., 2021), and physiological arousal (Horvers et al., 2024). To objectively measure cognitive load resulting from feedback frequency, we employ HR and HRV as physiological indicators. These measures have been widely recognized as reliable markers of cognitive load (Ayres et al., 2021; Blitz et al., 1970; Finsen et al., 2001; Thayer et al., 2012).

## 2 Hypothesizing the links

HR and HRV have long been established as key psychophysiological indicators of cognitive load (refer review Ayres et al., 2021; Thayer et al., 2012). The relationship between cognitive load and HR is positive (Ayres et al., 2021), whereas it is inverse with HRV (Ayres et al., 2021; Solhjoo et al., 2019). Cognitive load can be induced in an individual from various sources. Feedback is one such source (Lam et al., 2011). Several studies have analyzed the relationship between various feedback dimensions and cognitive load. For example, feedback type (Zheng et al., 2021), feedback valence (Redifer et al., 2021), and haptic feedback (Costa et al., 2019). However, the impact of frequent feedback on cognitive load using physiological measures is yet to be explored. Since feedback is considered to be a significant source of cognitive load, we propose the following hypothesis:

*Hypothesis 1*: There exists a significant mean difference in HR across the three conditions of feedback frequency.

*Hypothesis 2*: A significant mean difference in HRV exists across the three conditions of feedback frequency.

Feedback frequency has long been recognized as a key factor in influencing task performance (Chhokar and Wallin, 1984; Horvers et al., 2024; Ilgen et al., 1979; Lam et al., 2011). Traditionally, in performance management, it has been believed that frequent feedback results in better performance. For example, Mertens et al. (2021) conducted a field investigation that found evidence supporting the notion that increased feedback produces superior outcomes. Park et al. (2019) found that providing feedback more frequently had a more substantial impact on performance. Moreover, Kang et al. (2004) discovered that participants who received feedback after each session exhibited improved performance outcomes in a simulated job task. However, Lam et al. (2011) challenged the “more is better” concept, suggesting that optimal performance is achieved with moderate feedback frequency. Despite these findings, the evidence on how feedback frequency affects task performance remains inconclusive. Hence, we propose the following hypothesis:

*Hypothesis 3*: A significant mean difference in task performance exists across the three conditions of feedback frequency.

*Hypothesis 4*: Optimal performance in task is achieved when frequent feedback is provided.

Further, several studies have presented empirical evidence that performance is negatively related to HR (Chen et al., 2023; Solhjoo et al., 2019) and positively related to HRV (Forte et al., 2022; Luque-Casado et al., 2013; Maier and Hare, 2017; Schaich et al., 2020). Muthukrishnan et al. (2017) conducted a study that suggested a possible correlation between reduced HRV and impaired cognitive functions. However, it remains a question whether HR and HRV play a role in shaping the relationship between feedback frequency and task performance. Thus, we propose the following hypothesis:

*Hypothesis 5*: HR mediates the relationship between feedback frequency and task performance.

*Hypothesis 6*: HRV mediates the relationship between feedback frequency and task performance.

## 3 Methods

### 3.1 Participants

The study was approved by the Institute’s ethics committee. A theoretical evaluation was conducted through an experimental study employing a sample of 96 master’s students from an Indian university (*N* = 96). An email detailing the study requirements was sent to the eligible students, and 102 students initially agreed to participate. However, six students were excluded from the study due to incomplete/bad recordings. All participants possessed basic knowledge of arithmetic computations. A between-subjects approach was utilized, wherein participants were assigned randomly to one of three feedback frequency conditions. To establish symmetry, the distribution of individuals in each condition was balanced. A monetary incentive for the best performer of INR 5,000 (approximately $60) was introduced to encourage individuals to remain engaged and motivated throughout the tasks.

### 3.2 Experimental task

Participants were given an arithmetic test with a time limit that included addition, subtraction, and multiplication. An arithmetic test was used as a measure of task performance since it provides an objective way to obtain performance scores and requires focused attention and working memory (Musso et al., 2019). Additionally, arithmetic tasks are highly influenced by feedback (Peters et al., 2017), making it a reliable measure of task performance. The test was designed to be completed within a time limit of around 6 min using LabVanced (Finger et al., 2017). In this experiment, a query was displayed on the monitor for 3 s, after which there was a five-second gap for the participant to respond.

### 3.3 Procedures

The experiment was conducted in a controlled laboratory setting with a serene ambiance and stable temperature to enhance the precision of the findings. Before accessing the laboratory, every participant gave informed consent to participate in the study. Subsequently, participants were instructed to provide their demographic details. Further, the participants were directed to wear an E4 Empatica wristband. A three-minute relaxation interval was designated for assessing the baseline levels of HR and HRV. Data recording began at that point in time. Following the initial baseline period, each participant was equipped with a computer system and detailed instructions to aid them in effortlessly completing a mental arithmetic exam.

### 3.4 Device

The E4 wristband (Schuurmans et al., 2020) was used to measure HR and HRV. We used the Standard Deviation of NN Intervals (SDNN, in milliseconds) and Root Mean Square of Successive Differences (RMSSD, in milliseconds) based on the inter-beat interval (IBI) data extracted from the device as a quantifiable marker for HRV. The IBI files were artifacted by the device.

HR is a typically reported measurement and is measured as the number of beats in a period of time, most often reported per minute (Charles and Nixon, 2019). SDNN is the standard deviation of all normal RR (NN) intervals (Kleiger et al., 2005). RMSSD is the square root of the squares of the successive differences between NN intervals, essentially the average change in the interval between beats (von Neumann et al., 1941).

### 3.5 Feedback frequency

Participants were randomly assigned to one of the three feedback frequency conditions using a computer-generated randomization process. The randomization was implemented before participants began the study to prevent bias. The three conditions of feedback frequency are: Condition 1, where they received immediate feedback after each question, i.e., 20 times (high feedback frequency); Condition 2, where they received feedback after every set of five questions (moderate feedback frequency, given 4 times during the test, with each interval lasting 40 s); and Condition 3, where they received no feedback throughout the test (self-evaluation). The feedback information included the scores for correct answers out of the total questions.

### 3.6 Task performance

The overall performance score obtained from the arithmetic exam was used to evaluate each participant’s performance on specific tasks. Each correctly answered question earned one point, while erroneous responses received zero points. Each condition required the presentation of 20 questions. Thus, the maximum achievable score was 20, whereas the minimum achievable score was 0. The final scores were then converted into percentage scores and used for the analysis.

### 3.7 Control variables

We controlled for gender (1 = male; 0 = female) because prior research suggests that gender can influence how people process the feedback information and respond to it (Geddes and Konrad, 2003). Given that skin temperature is associated with cognitive and emotional processing (Ayres et al., 2021), we also controlled skin temperature to ensure the robustness and validity of our findings.

## 4 Results

An *a priori* power analysis was conducted using G*Power version 3.1 to determine the minimum sample size required to respond to our research questions (Faul et al., 2009). Results indicated the required minimum sample size to achieve 80% power for detecting a medium effect, at a significance criterion of *α* = 0.05, was *n* = 55 for Linear Multiple Regression: Fixed Model, R^2^ increase test.

The mean age of the participants was 25 years (*SD* = 3.44). Out of the total participants, 53 were males, and the remaining were females. As shown in Table 1, gender had a significant positive relationship with task performance indicating males performed better than females (*r* = 0.29, *p* < 0.01). Further, there exists a statistically significant negative association between HR and feedback frequency (*r* = −0.23, *p* < 0.05) and a positive association between HR and task performance (*r* = 0.25, *p* < 0.05). Further, HR had a statistically significant negative relation with both SDNN (*r* = −0.42, *p* < 0.001) and RMSSD (*r* = −0.44, *p* < 0.001). Finally, SDNN and RMSSD had a statistically significant positive association (*r* = 0.66, *p* < 0.001).

**Table 1:** Descriptives and correlation coefficients.

#	Variables	Mean	SD	1	2	3	4	5	6
1	Gender	0.55	0.5	–					
2	Skin temperature	32.43	1.36	0.29^**^	–				
3	HR	83.51	7.81	−0.00	0.10	–			
4	SDNN	62.40	21.90	−0.07	−0.17	−0.42^***^	–		
5	RMSSD	65.28	24.44	−0.18^+^	−0.41^***^	−0.44^***^	0.66^***^	–	
6	Feedback frequency	8.00	8.69	0.02	−0.07	−0.23^*^	0.20^+^	0.14	–
7	Task performance	0.54	0.22	0.29^**^	0.11	0.25^*^	−0.05	−0.10	−0.12

Gender: Male-1, Female-0. ^+^*p* < 0.10, ^*^p < 0.05, ^**^p < 0.01, ^***^p < 0.001.

**Figure 1:**
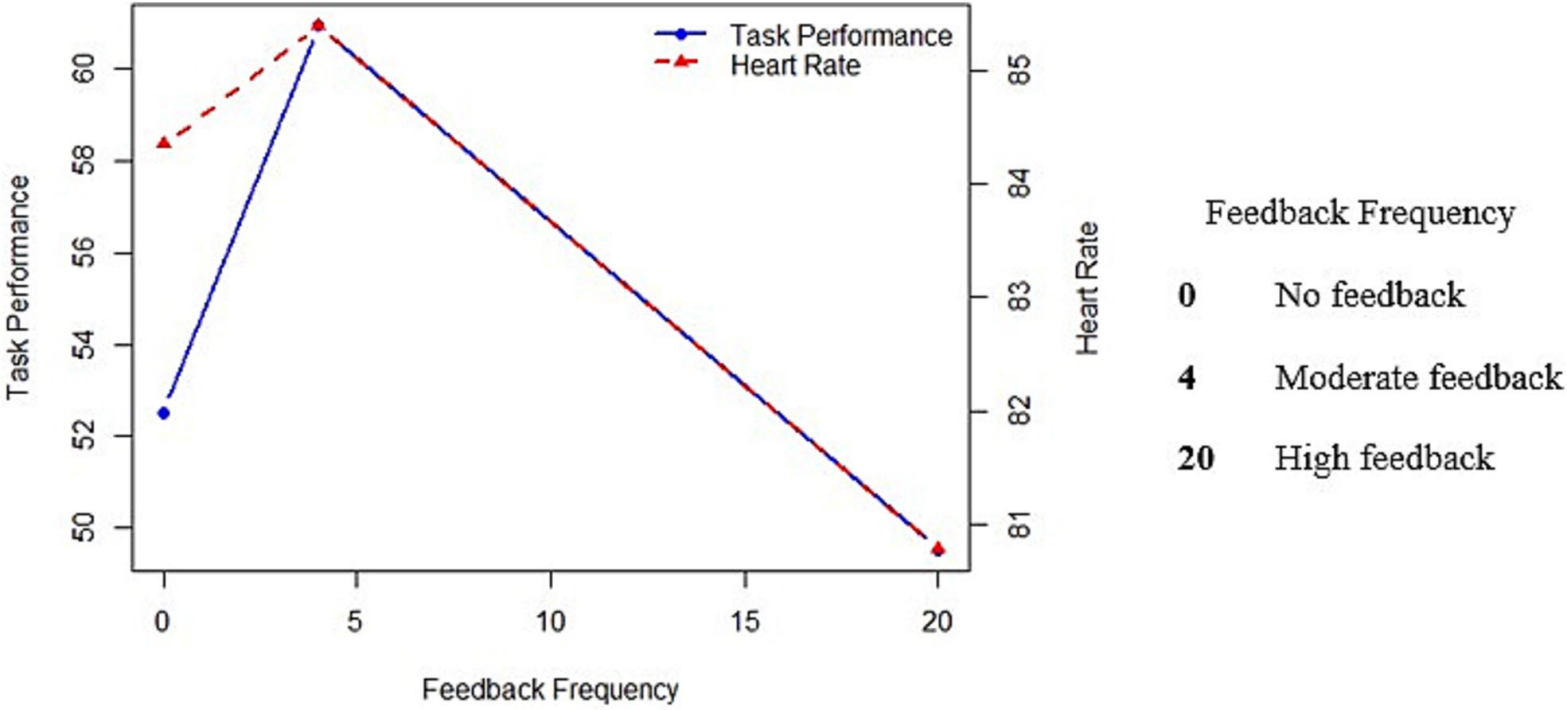
The mediating effect of heart rate on the relationship between feedback frequency and task performance.

Hypothesis 1 proposed that feedback frequency significantly affects HR across the three feedback scenarios. Using a one-way analysis of variance (ANOVA), we found that there was a significant impact of feedback frequency on the mean values of HR [*F*_(2,93)_ = 3.24, *p* < 0.05, *η^2^* = 0.07]. Thus, our hypothesis 1 is supported. Further, using the Bonferroni *post-hoc* test, we identified that the mean HR was highest under moderate feedback frequency and lowest when feedback was provided frequently. The same has been depicted in Figure 1.

Second, we proposed that the mean values of HRV will be impacted by the three conditions of feedback (hypothesis 2). We used one-way ANOVA to assess this hypothesis. We found that neither SDNN [*F*_(2,93)_ = 1.87*, p* = 0.16, *η^2^* = 0.04], nor RMSSD [*F*_(2,93)_ = 2.23*, p* = 0.11, *η^2^* = 0.05] was significantly impacted by the feedback frequency. Hence, we reject our second hypothesis.

In hypothesis 3, we proposed whether feedback frequency had a significant impact on the task performance of the participants. Using one-way ANOVA, we found no significant difference in the mean performance scores across the three feedback conditions [*F*_(2,93)_ = 2.419, *p* = 0.09, *η^2^* = 0.05]. Thus, we reject our hypothesis 3. Further, to test our hypothesis 4, indicating that optimal performance is achieved when feedback is provided frequently, we used the Bonferroni *post-hoc* test. We found that the optimal performance was achieved under moderate feedback conditions. However, the mean difference was not significant. Thus, we reject our hypothesis 4. In Figure 1, we can see that the relationship between feedback frequency and task performance is inverted-U.

In hypothesis 5, we posited that HR would mediate the association between feedback frequency and task performance. To test the mediation, we used regression analysis wherein, at first, we introduced HR as a dependent variable. Given the categorical nature of feedback frequency, dummy coding was utilized (*N*−1). The results revealed that moderate feedback frequency significantly caused HR to elevate (Table 2, Model 1, *β* = 4.32, *p* < 0.05). Also, no feedback caused HR to elevate; however, there was no significant impact (Table 2, Model 1, *β* = 3.61, *p* = 0.06). Subsequently, we introduced task performance as a dependent variable and incorporated HR into the model as a predictor variable, along with feedback frequency and control variables. We found HR to influence task performance positively (Table 2, Model 3, *β* = 0.01, *p* < 0.05), supporting the mediation effect (hypothesis 5). Refer to Figure 1 for the mediation effects of HR.

**Table 2:** Results of regression analysis for HR mediating the relationship between feedback frequency and task performance.

	HR	Task performance	
Variables	Model 1	Model 2	Model 3
Gender (1)	−0.30 (1.66)	0.12^**^(0.05)	0.13^**^(0.04)
Average skin temperature	0.39 (0.64)	0.00 (0.02)	−0.01 (0.02)
Feedback frequency (moderate)	4.32^*^(1.99)		0.09^+^(0.05)
Feedback frequency (no feedback)	3.61^+^(1.93)		0.01 (0.05)
HR			0.01^*^(0.00)
R^2^	0.10	0.08	0.18

The values in the bracket specify the standard error; Gender: Male-1, Female-0. ^+^*p* < 0.10, ^*^p < 0.05, ^**^p < 0.01.

Further, hypothesis 6 proposed that the relationship between feedback frequency and task performance will also be mediated by HRV. The regression method used to test hypothesis 5 was employed to test this hypothesis. However, as shown in Table 3, neither SDNN nor RMSSD were significantly influenced by feedback frequency. Thus, no mediation effect of HRV was found, leading to the rejection of hypothesis 6.

**Table 3:** Results of regression analysis for HRV (SDNN and RMSSD) mediating the relationship between feedback frequency and task performance.

	SDNN	Task performance	RMSSD	Task performance
Variables	Model 1	Model 2	Model 3	Model 4
Gender (1)	−1.52	0.13^**^	−3.44	0.13^**^
Average skin temperature	−2.38	−0.01	−6.53^***^	−0.01
Feedback frequency (moderate)	−6.83	0.12^*^	−7.28	0.12^*^
Feedback frequency (no feedback)	−9.65^+^	0.03	−5.18	0.03
SDNN		0.00		
RMSSD				0.00
R^2^	0.06	0.13	0.19	0.14

Gender: Male-1, Female-0. ^+^*p* < 0.10, ^*^p < 0.05, ^**^p < 0.01, ^***^p < 0.001.

## 5 Discussion

Prior research has shown that the presence of information or an increase in cognitive load elevates HR (Alshanskaia et al., 2024; Solhjoo et al., 2019). Consistent with this, our study found significant changes in HR due to changes in feedback frequency. Notably, the highest level of HR was observed under moderate feedback frequency, followed by the no feedback condition. Interestingly, optimal performance was also achieved under the moderate feedback frequency condition, aligning with the findings of Lam et al. (2011).

Furthermore, our study identified that feedback frequency causes fluctuation in HR, which, in turn, influences performance-highlighting HR’s mediating role in this relationship. However, our findings regarding the relationship between HR and performance differ from the previous literature. While prior studies suggest a negative association between HR and performance (Chen et al., 2023; Solhjoo et al., 2019), our results indicate a positive relationship. This positive association can be attributed to introducing feedback as a stimulus in this study.

Additionally, Kaegi et al. (1999) and Vrijkotte et al. (2000) suggested that the absence of feedback would lead to uncertainty and self-reflection, which can cause changes in emotional responses. Our results align with these studies, where heightened HR was observed in the case of self-evaluation (no feedback), indicating an increased cognitive load. When individuals do not receive feedback, they are ambiguous and uncertain about their performance, as indicated by Higgins et al. (1986), which leads to feelings of stress and tension, as described by Kim et al. (2018), further causing elevated HR (Solhjoo et al., 2019).

### 5.1 Theoretical contribution

This study contributes to performance management and psychophysiology by enhancing our understanding of the cognitive load caused by feedback frequency during task performance. Identifying the optimal feedback frequency as moderate, this study aligns with Lam et al. (2011). Additionally, it extends the literature on feedback frequency and performance by revealing that feedback frequency influences HR (an objective metric for cognitive load), which in turn significantly impacts performance.

Supporting CLT (Sweller, 2011), this study finds that cognitive load varies with feedback frequency, with the lowest observed under high feedback frequency. By employing an objective measure of cognitive load, this study overcomes the limitations of the previous studies (Mertens et al., 2021; Park et al., 2019; Lam et al., 2011) on feedback and cognitive load.

Though HRV is recognized as an objective measure of cognitive load (Ayres et al., 2021; Thayer et al., 2009), this study found no significant association between HRV and feedback frequency.

### 5.2 Practical implications

The findings of this study have several important implications for instructors or managers in educational and workplace settings, respectively. Given that moderate feedback frequency optimizes both HR and task performance, instructors or managers should consider implementing a balanced feedback mechanism to enhance individual development while minimizing cognitive load.

Additionally, the observed increase in HR under no feedback condition highlights the increased cognitive load caused by self-evaluation, suggesting that educational institutions and organizations should foster a feedback-rich culture to reduce uncertainty and improve individual well-being.

With the advent of AI-driven feedback systems, this study also highlights the need for researchers and designers in human-computer interaction to consider feedback frequency when creating environments that support cognitive efficiency and performance.

### 5.3 Limitations of the study and future research

Several limitations in the current study are important to note. The study uses an arithmetic test as a measure of task performance, and hence, the results may not be fully generalizable. The impact of information load under various feedback frequencies on HR and HRV might differ in the case of simulations, object/shape manipulations, or any physical activity due to varying cognitive demands on working memory. Although a relaxation period was provided before the experiment, participants were not screened for their psychological history, and its potential effects may not have been completely eliminated. Additionally, while the study aimed to induce cognitive load during task performance through an arithmetic test and feedback processing, the expertise of the problem solver or learner remains an important factor that cannot be overlooked. Moreover, the literature suggests various physiological factors besides HR and HRV work as an indicator of cognitive load (refer to Ayres et al., 2021). The same can be studied further by manipulating several other dimensions of feedback.

## 6 Conclusion

In summary, the research findings demonstrate that feedback frequency influences HR, with moderate feedback causing HR to elevate significantly, indicating an increased cognitive load. Moreover, the results demonstrated a positive relationship between HR and performance, with HR mediating the relationship between feedback frequency and task performance. Finally, HRV did not show a significant impact on task performance.

## Data Availability

The datasets presented in this article are not readily available because the data contain information that could compromise the privacy of research participants. Requests to access the datasets should be directed to p22ms006@iitj.ac.in.
